# Empathy, Mentalization, and Theory of Mind in Borderline Personality Disorder: Possible Overlap With Autism Spectrum Disorders

**DOI:** 10.3389/fpsyg.2021.626353

**Published:** 2021-02-11

**Authors:** Nicoletta Vegni, Caterina D'Ardia, Giulia Torregiani

**Affiliations:** ^1^Faculty of Psychology, Niccolò Cusano University, Rome, Italy; ^2^Niccolò Cusano University, Rome, Italy

**Keywords:** theory of mind, empathy, borderline personality disorder, autism spectrum disorder, mentalization

## Introduction

Autism spectrum disorder (ASD) is characterized by persistent deficits in social communication and interaction, behavior patterns, and narrow and repetitive interests or activities. Individuals with an ASD diagnosis have an atypical social approach to conversation reciprocity and a reduced sharing of interests, emotions, or feelings. Their verbal and non-verbal communication is poorly integrated, anomalies in eye contact and body language are present, as well as difficulties in understanding and using gestures (American Psychiatric Association, 2013). Over time the hypothesis has been confirmed that at the basis of the social compromises characterizing autism there are impairments in theory of mind (ToM), i.e., the ability to attribute mental states to people in order to explain and predict their behavior (Baron-Cohen et al., 1985; Baron-Cohen, 1995).

Some studies have related ASD to personality disorders, as shown by recent reviews of the literature, highlighting that there is a percentage of comorbidity between the autistic spectrum and psychopathological traits (Matson and Nebel-Schwalm, 2007; Matson and Goldin, 2013; Mannion et al., 2014). In relation to the analyses found in the literature, the disorder that in our opinion shows particular possible overlaps with ASD in terms of lack of empathy and theory of mind seems to be BPD.

Borderline personality disorder (BPD) is characterized by pervasive disadaptive modes of thought and behavior. BPD, as stated in DSM-5 (American Psychiatric Association, 2013), presents a pervasive pattern of interpersonal relationships, self-image and mood instability, and a marked impulsiveness, which generally begins in early adulthood in different contexts of daily life. Its distinctive features include emotional dysregulation, fear of neglect, malfunctioning in interpersonal relationships, fractioned thinking, and difficulty in impulse control.

Many studies have been conducted to identify the components of borderline personality disorder, including emotional dysregulation (Yen et al., 2002; Conklin et al., 2006), attachment (Fonagy, 2000), and theory of mind (Harari et al., 2010; Franzen et al., 2011). Emotional dysregulation is known as one of the main symptoms in patients with borderline personality disorder. Many studies have found that many borderline personality traits are the result of emotional dysregulation (Barnow et al., 2012; Ghiasi et al., 2016; Salgado et al., 2020). Studies have also revealed that people with this disorder have problems identifying, distinguishing, and integrating their own emotions with those of other individuals (Harari et al., 2010).

Recently, many have begun to consider empathy as a construct composed by two components, one cognitive and one affective (Baron-Cohen and Wheelwright, 2004; Shamay-Tsoory, 2011). Affective empathy involves the experience of the feelings and emotions of others through recognition, sensitivity to the emotions of others, and sharing the emotional experiences of others through an affective response appropriate to the situation of the other (Batchelder et al., 2017).

Cognitive empathy involves the process of understanding another person's perspective by adopting another person's point of view. The ability to adopt another person's point of view is consistent with the concept of theory of mind (ToM). Cognitive empathy also includes the ability to judge and understand the intentions of others in order to monitor one's own intentions (Batchelder et al., 2017).

This paper presents some opinions drawn from the analysis of literature from the last 20 years which has analyzed the link between ASD and personality disorders, and specifically BPD. The articles were selected according to the time criterion of the year of publication through the PUBMED database.

Papers that included the following keywords were considered: “borderline personality disorders and empathy,”. “borderline personality disorders and theory of mind,” “borderline personality disorders and autism,” “autism and empathy,” and “autism and theory of mind.” Papers published between 2000 and 2020 were included.

The only articles outside the timeframe that were included were related to theoretical constructs.

## Overlaps Between BPD and ASD

Difficulties related to the social and relational fields are characteristics found in patients with BPD, as well as in patients with ASD. Considering patients with high functioning autism, Hofvander et al. (2009) showed that 68% of the sample, composed of adults with ASD, met the criteria for at least one personality disorder. In accordance with this, when checking for possible personality disorders in a group of young adults with Asperger's syndrome, a study showed a considerable overlap of symptoms between Asperger's syndrome and some personality disorders (Lugnegård et al., 2012). Baron-Cohen et al. (1985) emphasizes how the social and communicative difficulties of ASD subjects can be traced back to a deficiency of maturation of the theory of mind (ToM), or rather, of the cognitive mechanism responsible for the analysis of one's own and others' mental states, as well as highlighting in these subjects important difficulties in terms of empathy. Considering empathy as the ability to understand another's state of mind and systematization as the ability to perceive the models of change (the rules) that allow us to understand how things work and predict their future, some authors (Wheelwright et al., 2006) have developed a theory according to which different brain types are attributed to individuals according to their tendency to be more empathic or systematic.

Baron Cohen compared ASD with BPD in terms of lack of empathy and specifically with a “zero degree of empathy” measured with the Empathy Quotient (EQ) (Wheelwright et al., 2006; Baron-Cohen, 2011) as both disorders would manifest difficulties in social and interpersonal interactions as discriminating symptoms.

As for systematization ability, measured by the Systematization Quotient (SQ) (Goldenfeld et al., 2005; Wheelwright et al., 2006) patients with BPD would show low levels while patients with ASD would show higher levels than the average of normo-typical subjects. This difference would explain the tendency of ASD subjects to follow rules and the tendency to deviance and take risks in BPD subjects (Baron-Cohen, 2011). The study conducted by Dudas et al. (2017) analyzed a sample of subjects, including ASD, BPD, double-diagnosed, and normo-typical (NC) subjects compared according to EQ and SQ indices. In the SQ-R measure, all clinical groups had a statistically significant score that was higher than the control group. These data, which show high levels of systematization in patients with BPD as well as in patients with ASD, seem to contrast the idea that it is precisely systematization that differentiates BPD from ASD in relation to the tendency of the former to take risks and be impulsive and therefore to place themselves within a “spectrum of empathy” equal to negative zero rather than positive (Baron-Cohen, 2011).

It has been clarified in the literature that a lack of empathy is a hallmark of ASD (Harmsen, 2019; Stroth et al., 2019).

At the same time, scientific literature presents various evidence in favor of empathic and mentalization deficits in BPD, which can be interpreted as possible causes of social and relational problems. Baron-Cohen describes the empathic difficulties of patients with BPD, in relation to the recognition and response components, describing them as deficient in reacting to others with an appropriate emotion (empathic response), and with difficulty in effectively and precisely determining the intentions and emotions of others' facial expressions (Baron-Cohen, 2011) In a study, how ambiguity of stimuli can lead to a reduction in cognitive or emotional empathy in BPD was discussed (Niedtfeld, 2017). Video clips were shown to 34 patients with BPD and 32 controls, through which vocal content, prosodies, and facial expressions were presented. BPD patients showed greater emotional empathy when the proposed stimuli included emotions expressed in a non-verbal way, while with regard to cognitive empathy, there were no significant differences between BPD and controls. These results suggest that subjects with BPD show altered emotional empathy, experiencing higher rates of emotional contagion when emotions are expressed non-verbally. The latter may contribute to misunderstandings and inappropriate social behavior. Differences in amygdala activation in subjects with BPD compared to control subjects were also found in a study revealing difficulty in understanding neutral facial expressions, often interpreting them as threatening (Donegan et al., 2003). Regarding the analysis of the different components of empathy involved in BPD, using a self-report measure of empathy, the Interpersonal Reactivity Index (IRI; Davis, 1983), a study showed that women diagnosed with BPD showed higher average levels of affective empathy and lower average levels of cognitive empathy, compared to a control sample of women with anorexia nervosa and an undiagnosed control group (Guttman and Laporte, 2002). These results were confirmed by another research that demonstrated a “double dissociation” of cognitive and affective empathy in BPD, suggesting that the behavioral difficulties manifested in BPD can be explained by a dysfunctional model of empathic capacity (Harari et al., 2010). The authors started from the hypothesis that the interpersonal malfunction typical of BPD would find justification in their low levels of cognitive empathy and theory of mind, while higher levels of affective empathy would account for emotional hyperactivity.

To evaluate the cognitive and affective aspects of empathy IRI was used, while the faux pas recognition test was adopted for measurements related to the theory of mind (Phillips et al., 1998). The results found that the controls had higher IRI scores in the cognitive component of empathy than in the affective component, whereas the BPD group showed the opposite pattern. Significant differences were shown between BPD patients and the control group in the understanding of the theory of the cognitive mind but not in the emotional one, detected with the faux pas recognition test. In the study BPD patients showed worse performance in both cognitive empathy and cognitive mind theory measures, but there were no differences in the understanding of emotional mind theory, while the affective aspects of empathy were even better in patients with BPD. Providing support in terms of neurobiological comparison to the “dissociation” of cognitive and affective empathy in BPD, a study, including fMRI data, showed that patients with BPD compared to controls showed lower activation during cognitive empathy activities in the upper temporal fissure (STS), an area associated with the inference of other people's mental states (Zaki et al., 2009), but greater activation in the medium insular cortex (Jackson et al., 2006), an area associated with personal distress, during activities involving emotional empathy (Dziobek et al., 2011). Moreover, considering the typical manifestations of the borderline personality, two processes were put in relation to empathic dysfunctions: emotional dysregulation, i.e., highly uncontrolled emotional reactivity, an inability to modulate internal emotions, resulting in an inappropriate emotional and behavioral response (Linehan, 1993; Gratz et al., 2006) and hyper-mentalization, i.e., a misinterpretation or a sort of super reference of one's own and others' thoughts and feelings (Sharp et al., 2011, 2013). Another study examined the relationships between emotional dysregulation, hyper-mentalization, and cognitive and affective empathy in 252 adolescent patients, divided into BPD subjects and undiagnosed subjects, revealing that in both groups, emotional dysregulation was related to increased affective empathy. Hyper-mentalization, on the other hand, was correlated with reduced cognitive empathy in patients with BPD, while hyper-mentalization was not correlated with any kind of empathy in patients without BPD (Kalpakci et al., 2016). As regards, difficulties in mentalization or the ability to precisely infer the mental states of others, a dysfunctional mechanism of these abilities was hypothesized at the basis of borderline personality disorder (Fonagy et al., 1991; Sharp and Fonagy, 2008). Moreover, with the aim of examining the mediating role of the regulation of emotions in the relationship between ToM and borderline traits in adolescents, a study found a trend that associates an overall reduced ToM capacity with an increase in borderline traits and a consistent correlation between these and difficulties in emotional regulation; not an absence of mentalization capacity but an over-mentalization (Sharp et al., 2011). This hyper-mentalization would manifest itself as an excessive interpretation of the mental state of others. The tendency to make overly complex inferences about social signals that have been found to be erroneous has also been shown. These subjects would tend to over-interpret social signals by overestimating them. These results suggest that the difficulties of emotional dysregulation, at least in part, mediate the association between hyper-mentalization and BPD.

## Discussion

It should be clarified that there are often overlaps in literature between the different cognitive and affective components of empathy and mentalization, and there is therefore no unanimous consensus on this issue. Moreover, some authors understand the theory of mind in terms of the “presence or absence” of this ability with the consequent effective functioning or not of the psychic activities linked to the attribution of mental states; others consider it as part of a wider mental activity, i.e., metacognition, which would include, in addition to ToM, more sophisticated mentalization abilities. It seems, therefore, that common characteristics of serious personality disorders can be traced back to alterations in metathought and emotional regulation, both of which in turn are expressions of metacognitive functions. Each particular disorder is characterized by a specific profile of impairment (more or less serious) of some subfunctions of metacognition. In particular, the borderline disorder as opposed to autism would be characterized by a difficulty to distinguish between representation and reality, to modulate one's mental states functionally and adaptively, and to integrate the various elements of mental activity (sensations, emotions, thoughts, etc.) into continuous and coherent narrations of oneself, others, and the world.

The studies presented have uniformly shown that the lack of empathy and theory of mind is an overlapping aspect in BPD and ASD but there is still little evidence regarding the differences between the two clinical domains with respect to the affective and cognitive components of empathy itself. Furthermore, in the studies, analyzed measurements are carried out with different instruments and tests, which means that the results of one study cannot be fully compared with those of another.

It would be useful, also with regard to the development of possible integrated therapies, to investigate the link between BPD and ASD in terms of lack of empathy, ToM, and mentalization using shared protocols for diagnosis that can allow homogeneous measurements that do not leave room for the interpretation of individual scores.

Finally, it would be interesting to investigate possible overlapping between BPD and ASD with respect to executive functions and impulse control, an aspect not yet particularly stressed in the scientific literature.

## Author Contributions

NV: developed the topic of the work and carried out the final linguistic review. CD'A: contribution for the ASD topic. GT: references research. All authors contributed to the article and approved the submitted version.

## Conflict of Interest

The authors declare that the research was conducted in the absence of any commercial or financial relationships that could be construed as a potential conflict of interest.
